# Memorable Experiences with Sad Music—Reasons, Reactions and Mechanisms of Three Types of Experiences

**DOI:** 10.1371/journal.pone.0157444

**Published:** 2016-06-14

**Authors:** Tuomas Eerola, Henna-Riikka Peltola

**Affiliations:** 1 Department of Music, University of Jyväskylä, Jyväskylä, Finland; 2 Department of Music, Durham University, Durham, United Kingdom; University of Zurich, SWITZERLAND

## Abstract

Reactions to memorable experiences of sad music were studied by means of a survey administered to a convenience (N = 1577), representative (N = 445), and quota sample (N = 414). The survey explored the reasons, mechanisms, and emotions of such experiences. Memorable experiences linked with sad music typically occurred in relation to extremely familiar music, caused intense and pleasurable experiences, which were accompanied by physiological reactions and positive mood changes in about a third of the participants. A consistent structure of reasons and emotions for these experiences was identified through exploratory and confirmatory factor analyses across the samples. Three types of sadness experiences were established, one that was genuinely negative (Grief-Stricken Sorrow) and two that were positive (Comforting Sorrow and Sweet Sorrow). Each type of emotion exhibited certain individual differences and had distinct profiles in terms of the underlying reasons, mechanisms, and elicited reactions. The prevalence of these broad types of emotional experiences suggested that positive experiences are the most frequent, but negative experiences were not uncommon in any of the samples. The findings have implications for measuring emotions induced by music and fiction in general, and call attention to the non-pleasurable aspects of these experiences.

## Introduction

The experience of sadness in musical listening is a conundrum that has been pondered by numerous scholars. The phenomenon presents seemingly conflicting emotional experiences: music typically induces a range of positive emotions [1] but sadness as an emotion is considered in psychology to be negative [2]. Sadness is an emotion commonly associated with music [1], in which context it is often paradoxically related to pleasurable experiences [3–5]. Surveys not involving music listening suggest that people link sadness with a wide range of emotions [6–8], including nostalgia, peacefulness, and tenderness, while lab-based listening experiments portray a similar multifaceted picture of sadness in music [9]. Yet, music-induced sadness has been shown to generate similar negative biases in cognitive processing to those produced by real, autobiographically-induced sadness [10], and the emotions induced by sad music can be discriminated from several other emotions through psychophysiology [11] including from liking using neural responses [12]. Most cultures have a special role for music (laments, funeral songs) used in the contexts of mourning and loss [13–17]. Such examples suggest that sad music may be linked with actual negative experiences in addition to the positive experiences often cited. Indeed, a recent qualitative study of Western listeners found out a range of intense experiences relating to grief and low-intensity depression to be associated with to music-induced sadness [18], and also offered a tentative typology of such experiences (*grief*, *melancholia*, and *sweet sorrow*).

One possibility is that studies involving music and sadness may not differentiate the emotions experienced in sufficient detail, thus conflating inherently incompatible emotional experiences that have distinct mechanisms, outcomes and reasons. Failure to address the key moderating variables such as the focus of the emotions (expressed or experienced), the emotion-induction mechanisms [11], (particularly the role of the episodic memories, or selection and familiarity with the music [19]), may have led the field to consider only a limited range of emotional responses to sad music: responses from individuals who actually enjoy and derive pleasure from it.

Here, we aim to clarify the relevant reasons, mechanisms, and emotions involved in musical experiences associated with sadness. Although Taruffi and Koelsch [6] have recently provided an overview of the contents of the emotions related to sad music, we are still far from finding the answers to the basic questions: their study focussed on generic semantic aspects of the experiences instead of episodic memories, but the latter are known to be richer than semantic knowledge [20]. The study, focussing only on the rewarding aspect of listening to sad music, utilised a generic self-report instrument on emotions (the *Geneva Emotion Music Scale*, GEMS) that contains predominantly positive and aesthetic emotions without direct utilitarian functions [21], which might have prevented their participants from providing nuanced details of emotions. This is particularly problematic since the study showed that the most prevalent type of situation in which participants chose to engage with sad music was that titled *emotional distress* (breakup, grieving, etc.), examples of which are likely to involve a range of negative emotions, as suggested by an in-depth analysis of free responses relating to participants’ engagement with sad music [18]. These analyses have suggested that strong and clearly negative emotions such as fear, anger and grief are not marginal emotions in the context of sad music, particularly when the music is linked with autobiographical events and associations. Moreover, the sample in the study by Taruffi and Koelsch [6] was an uncontrolled internet sample, leaving issues of prevalence, self-selection bias and expertise unanswered.

Our aim is to elucidate what are the general characteristics of music-induced sadness. More specifically, we aim to answer the following questions:

1. What are the characteristics of music-induced sadness in terms of the situations, changes in physical and mental states and physical reactions?
2. What are the dominant psychological mechanisms and reasons behind the experiences involving sad music?
3. What are the typical emotions associated with such experiences and the specific structure underlying these?
4. How prevalent are the different types of experiences previously identified in association with listening to sad music?

These questions were considered to be best explored through a structured survey, using both existing instruments and a new series of questions derived from previous findings on music and sadness. We differentiated between generic and specific questions, the latter referring to a specific memorable example that the participants are asked to specify, since this is known to provide more accurate and definitive information than generic questions [20]. Collecting data from different samples enabled us to carry out exploratory and confirmatory factor analyses of the main conceptual structures (reasons, mechanisms, and emotions), and offered a better estimation of the prevalence and relevance of these themes for sadness in music.

## Methods

### Survey items and procedure

University of Jyväskylä Ethics Committee approval was obtained for the study. The data collection was anonymous and carried out online which meant that no full informed consent statements were signed by the participants, although a statement at the beginning of the survey explained the voluntary nature of their participation and the use and handling of the data.

In addition to basic background information of the respondents (age, gender, education, interest in listening to music, and how often they listen to sad music) a number of key themes were identified for the survey. Sad music was defined as “music that can be described as sad by the listener; sad-sounding, sad atmosphere in the music, music disseminates sad narrative, etc.”

The first section dealt with broad attitudes towards sad music, implemented through an existing instrument, *Attitudes towards Sad Music* [22] that assesses six different types of attitudes linked with engaging in sad music. The rationale for employing this instrument was to establish links between the attitudes towards sad music and the memorable sad music experiences.

The second section explored the importance of 24 reasons to listen to sad music, as well as the emotional functions of listening to sad music, derived from previous studies [6, 19, 22]. These 24 reasons, shown in Table 1, were provided as checklists from which the participant could choose all that applied.

**Table 1:** Loadings of the most frequent reasons for listening to sad music across the three rotated factors (loadings below 0.32 are not displayed).

Reason (I listen to sad music to…)	PC1	PC2	PC3
… to channel my emotions	0.71		
… to get in touch with my emotions	0.70^*α*^		
… to reminisce about past events/places/people	0.66		
… to get comfort (music acts as a supportive friend)	0.63^*α*^		
… to re-experience feelings I have experienced before	0.62^*α*^		
… to match my emotional state with the music	0.50		
… to feel closer to my loved ones	0.45		
… to sort my thoughts	0.45		
… to share my feelings induced by music with others		0.68	
… to reveal my musical preferences to others		0.68^*α*^	
… to let the music express thoughts to others		0.68^*α*^	
… to feel connected with the other(s)		0.65^*α*^	
… to experience sense of belonging to a community		0.55	
… to calm down and relax			0.70^*α*^
… to empathize with the expressive quality in the music			0.65^*α*^
… because I find it beautiful			0.65
… to enhance my mood			0.53
… to empathize with the story conveyed by music			0.43^*α*^
… to experience new feelings			0.41
*Alpha*	0.86	0.81	0.82
*Loadings*	2.96	2.19	2.16
*Variance explained*	16%	12%	11%

Table notes. Items indicated with ^*α*^ belong to the simplified CFA based on the three highest alpha values within the factors.

The third section focussed on a participant’s chosen memorable sad music experience. This question was modelled after *Adult Crying Inventory* by Vingerhoets and Cornelius [23] and was designed to probe the situation, feelings, functions, and mechanisms involved with music-induced sadness using an episodic memory rather than relying on generic semantic memories that often have biases [20]. First, the participants had to briefly describe the situation, then provide answers to structured questions about the situation, chronology and duration, music choice, motivation for choosing to listen to sad music, familiarity with music, autobiographical relevance, and the emotions or feelings experienced during the episode. For the last question, the participants could choose terms from a list of 36 emotion terms relevant for music and sadness that was partly derived from previous research [18, 21] and supplemented with terms from pilot studies.

The participants were also asked to nominate the relevant emotion mechanisms involved using a 10-item checklist [24]. Furthermore, answers to a short series of questions about participants’ mental and physical reactions before and after the episode were collected, and they were requested to indicate the overall intensity and pleasantness of the experience using 7-point likert scales. Finally, based on a previous study that examined both negative and positive emotions experienced in relation to sad music [18], we asked the participants to rate the frequency of three broad categories of emotion using a scale from 1 to 5 (1 = never, 2 = rarely, 3 = sometimes, 4 = frequently, 5 = very frequently). These broad emotion categories were as follows: *grief*, consisting of deep hatred, grief, or loss; *sadness* consisting of depression, melancholy and apathy; and *relief* consisting of comfort, relief, fulfilment, and feelings of elation.

The survey was implemented in an online service, in which the order of the items within the sets of questions was randomised. Participation was voluntary and anonymous, although the participants could leave an e-mail address if they wished to participate in a prize draw including two 50-euro vouchers.

The survey instruments were identical for all samples but were translated into English (except for *Attitudes towards Sad Music* where the original English version was used) first by authors and an independent back translation was carried out by a native speaker of both languages. Also minor changes made to background questions relating to education, income and geographical location. The items within the questions were randomly ordered for each participant but the order of sets of questions remained the same (attitudes, reasons, and memorable experience).

### Samples

Three samples were used in the present study. The first was a convenience sample from a single country (Finland), hereafter S1. The second was a representative sample of the UK population to qualify and expand the findings of the convenience sample (S2). The third sample (S3) utilised quota sampling of the Finnish population to provide a selection of listeners similar to the representative UK sample in terms of gender and amount of listening to sad music in the representative of UK sample. The purpose of this was to facilitate the comparison between the two countries. A numerical summary of the samples S1-S3 is given in S1 Table.

**Convenience sample (S1)** A convenience sample was utilised since the aim was to obtain a large number of responses on this specific topic. The national media in Finland agreed to circulate the call for the survey. This is a similar recruitment technique to that used in previous studies [6, 8], and has the potential problem of appealing particularly to people who are likely to be positive about music or music and sadness. 1596 participants completed the survey, out of which 71% were women, and this proportion was constant across age range (18-74, M = 36.08 SD = 12.71). The participants were better educated than the Finnish population, 58.8% of the participants possessed BA/MA equivalent or higher level education (29.3% in Finland), and 21.7% reported Basic education or less (30.6% in Finland). On average, the participants were highly interested in music; 19.4% classified themselves as non-musicians, 40.7% as music lovers, 31.1% as amateur musicians, 5.5% as semi-professionals, and 3.3% as professionals. Most participants listened to music at least once a day (42.6%), or several times a day (42.4%), or multiple times a week (11.3%). The frequency of participant’s experiences of listening to sad music is shown in S1 Table, which support the notion that these participants do engage in listening to sad music (47.4% often or frequently).

**Representative sample (S2)** To explore the reliability and generalisibility of the findings of the convenience sample, the same survey was administered to a representative sample from a different country. A stratified sample of UK citizens was taken, in which region, age and gender formed the individually controlled strata. The sample was obtained from *SurveyMonkey Audience* (www.surveymonkey.com/mp/audience), and consisted of 445 participants. 53.3% of the sample were women, and age range followed the UK age distribution (18-24 9.19%, 25-34 17.26%, 35-44 22.42%, 45-54 25.56%, 55-64 22.87% 65-74 2.24% 75+ 0.45%). The frequency of listening to sad music by the representative sample is shown in Supporting Information (S1 Table).

**Quota sample (S3)** The third sample utilised quota sampling to create a sampling in which there were equal numbers of each gender and those listening to sad music (5 categories) as in the representative UK sample. The purpose was to seek a balanced representation of men and women who would not be especially keen on music and sadness in order to compare the findings with other samples that will vary in this respect. This data collection was, of course, carried after the representative (UK) sample was made.

As expected, the S1 and S2 differed in terms of several background variables such as age distribution (*χ*^2^(5) = 146.6, *p*<.001), gender (*χ*^2^(1) = 71.4, *p*<.001) and the frequency of listening to sad music (*χ*^2^(4) = 215.8, *p*<.001), see S1 Table for details. However, the S3 sample was not different from the S2 with respect to gender (*χ*^2^(1) = 0.56, *p* = 0.46) and frequency of listening to sad music (*χ*^2^(4) = 7.6, *p* = .11). The S2 and S3 were inherently different, since they were collected from different countries, and were sampled in a different fashion. Hence, the details in the samples did not match (for example, there is an age difference between the S2 and S3, *χ*^2^(5) = 62.5 *p*<.001). However, using such separate samples allows us to disentangle the contribution of the sample differences to the results. We will explore other sample related differences more closely within the main research questions.

## Results

The analysis strategy consists of providing descriptive summaries of the main questions with all samples. The structure discovery of the reasons and emotions was carried out with Exploratory Factor Analysis (EFA) using the largest sample (S1) after which the identified structures were tested with the two other samples using Confirmatory Factor Analysis (CFA).

In the S1, outlier screening revealed four participants that gave flat responses to the 25-item ASM instrument with 7-point likert scales, and these individuals were eliminated. Another fourteen participants had a reverse (*r* > -.40) response pattern to the mean ratings in the same instrument, suggesting a possible confusion in the labelling of the statements. These participants were also discarded, leaving 1577 participants in the analysis.

For the S2 (representative sample from UK) and the S3 (quota sample from Finland), no outliers were found in the screening stage. It is also worth pointing out that the EFA and CFA analyses were carried out with binary data (choices of emotions, reasons, and mechanisms) using polychoric correlations which has been shown to be less prone to produce over-dimensionsalisation than product-moment correlations [25], and we also employ simulation and optimisation for factor retention in EFA [26] to avoid overfitting.

### Reasons for engaging with sad music

The 24 statements related to reasons for engaging with sad music yielded a different overall number of reasons across the samples (an average of 9.95 reasons in S1, 4.62 in S2, and 8.33 in S3). The rankings of the reasons also vary between the samples, but there is an overall agreement of the most important reasons. Within all samples, the top five reasons include *To listen to music privately* (#2, #1 and #1 for S1, S2 and S3 samples, respectively), closely followed by *beauty of the music* (#1, #2, #3), and *to get comfort* (#3, #4, and #5 reason across samples), and *to reminisce* (#5, #2, #5). It is also apparent that some reasons were infrequently chosen, including those relating to sharing emotions or choosing sad music because it produces feelings of belonging. A full list of the ranked frequency of the 24 statements related to reasons for engaging with sad music across the three samples is given in Supporting Information (S2 Table).

Looking at frequency of reasons and background variables, gender seems to play a large role here. Several themes under reasons were more likely to be nominated by women in all samples; Women tend to favour statements relating to reminiscing (*χ*^2^ = 21.0, *p*<.001), as well as social and nurturing reasons such as comforting (*χ*^2^ = 22.8, p<.001), channeling (*χ*^2^ = 12.5, *p*<.001), to be closer to loved ones (*χ*^2^ = 10.4, *p*<.001), or to share emotions with others (*χ*^2^ = 12.6, *p*<.001). In comparison to women, men favour strategies that involve experiencing new emotions (*χ*^2^ = 12.0, *p*<.001), or sharing music preferences (*χ*^2^ = 12.6, *p*<.001), which possibly highlight the more cognitive reappraisal strategies in general [27].

To explore the possible structure within these statements, the factorability of responses across the statements was conducted using the polychoric correlations and carrying out the EFA with the largest dataset (S1). Healthy Kaiser-Meyer-Olkin measure of sampling adequacy (0.84) was obtained. Subsequently, Velicer’s MAP reduction algorithm, which is one of the most robust ways to determine to determine the number of components [28], was utilised to find the optimal number of components to extract. This offered 3 components and the factor analysis with oblimin rotation was utilised to increase the interpretability of the loadings. This model explained 36% of the variance and obtained a decent fit to the data (RMSR = 0.07).

Improvements were examined by looking at the items obtaining loadings below 0.32. This led to removal of three items (“to avoid negative thoughts/feelings”, “to gain a more realistic perspective”, and “other”). This improved the model slightly (*χ*^2^186 = 1234.2, CFI = 0.824, RMSEA = 0.057). Next, the items containing high cross-loadings were eliminated using alpha factoring criterion (item alphas < 0.60 within each factor are eliminated). This removed two conflicting items, (“I want to listen privately”, “I want to be closer to my loved ones”) and yielded a more parsimonious model (*χ*^2^101 = 689.2, CFI = 0.869, RMSEA = 0.058). This pruned version of the model is shown in Table 1. The overall variance explained remained modest (39%) due to the binary nature of the data. The first reason could be labelled as *Reflection*, the second factor as *Belonging*, and the third factor as *Relaxation*. The contents of the three-factor structure that was consistently rated across the participants is shown in Table 1. It is worth observing that several putatively interesting explanations such as “to gain more realistic perspective” [29] lie outside of the main reasons given for listening to sad music. It should be acknowledged that listeners may not be able to articulate all of the reasons they have for listening to sad music, and, therefore, our list, though culled from a range of past studies, is not guaranteed to be comprehensive.

CFA of the final three-factor model obtained with the S1 with S2 produces a plausible fit with the data (*χ*^2^(167) = 334.3, *p*<.001, CFI = 0.886, RMSEA = 0.047, CI_90_ 0.040-0.055) and CFI = 0.779, RMSEA = 0.071, CI_90_ 0.064-0.078 with S3. Again, the confirmatory model seems to be overspecified in terms of the incremental fit index (CFI) that penalises the fit for a high number of parameters. To simplify the model whilst retaining the identified structure, the three items scoring the highest alphas within the factors was taken from the S1. This model, consisting of 3 factors with 3 items each (indicated in the Table 1 with ^*α*^ labels), yielded a good fit for S2, *χ*^2^(24) = 53.2, *p*<.001, CFI = 0.937, RMSEA = 0.052, CI_90_ 0.033-0.071 and an excellent fit for the S3, *χ*^2^(24) = 38.7, *p*<.05, CFI = 0.969, RMSEA = 0.039, CI_90_ 0.012-0.060. Thus, the simple three factor structure seems to account for the responses of all samples.

To explore the notions behind the factors underlying the reasons for engaging with sad music in more detail, the responses to the specific statements associated with theory-driven list of six categories of affects (pleasure, nostalgia, hurt, melancholia, comfort, and pain) are shown in S3 Table. For broadly positive emotions (pleasure, comfort, nostalgia), the most frequently nominated reasons are similar to the general reasons outlined in S2 Table, consisting of beauty, reminiscing, and relaxing. For the negative emotions (hurt, pain, and melancholia), which were generally less frequently nominated, the pattern of the most often mentioned reasons is perhaps more interesting, since it highlights the importance of personal losses and being reminded of loved ones that have passed away, emphasising that music often conveys a tragic or hopeless narrative.

To expand the reasons underlying choosing to listen to sad music, the relevance of various mechanisms responsible for music-induced emotions were explored. The list of mechanisms, though largely derived from Juslin and Västfjäll [30], also included additions such as beauty of the music [24], sharing emotions with others, and expectations of re-experiences the emotion. It is clear that the emotional expression of the music itself (#1, #2, and #1 ranked reason for S1, S2 and S3), the beauty of the music (#2, #3, and #2), and memories (#4, #1, and #3) are the most often implicated mechanisms. These have already been implicated in several studies [6, 10, 19]. Interestingly, most of the mechanisms linked with music itself such as surprising events, strong captivating rhythm or expectations about how it will unfold, seem to play a relatively minor role here (from 6 to 16 percent of the nominations across the samples), as can be seen in summary provided in S3 Table.

To simplify the list of ten mechanisms for the follow-up analyses, the mechanisms were subjected to principal component analysis with the largest sample (S1) using the same parameters as the analysis of reasons and affects (polychoric correlations, promax rotation with the elimination of loadings under .32). This yielded a three-component structure that explained 43.3% (*χ*^2^ = 2336, *p*<.001, RMSR = 0.14) of variance in the nominations of the mechanisms in S1. In the model, visual imagery, which did not fit any of the components, was eliminated, but otherwise the model provides an intuitively plausible reduction of the separate mechanisms into three broad ones, as shown by the last column in S3 Table.

### Typical reactions to memorable experiences with sad music

After the generic and unspecific questions about attitudes towards sad music and reasons involved in listening to sad music, the remainder of the questions addressed a memorable experience with sad music.

Turning now to the largest sample, the open responses of the participants were analysed using a *thematic content analysis* by one of the authors (HP). This is a qualitative analysis method for identifying and analysing patterns within textual data [31]. The analysis of the present study represents *critical realism paradigm*, which aims to explain and make objective predictions about reality, but recognises that objective reality is only imperfectly comprehendible [32]. The aim of the analysis was to provide an accurate semantic description of the entire data set in order to explore the content of the predominant themes.

The initial inductive analysis resulted in 11 thematic categories that contained differing amount of sub-categories. A 10% random sub-sample of these textual responses was recoded using the proposed thematic categories by another author (TE) unfamiliar with the initial coding. This resulted in 92% match in the thematic categories, suggesting a credible content validity of thematic assignment of the open responses. After a negotiation between the coders, the final identification of nine themes took place. A proper reporting of the content and meaning of each theme is beyond the scope of the present article, but a short description of the prevalence of important themes is provided below.

The descriptions of the situations indicated that these experiences were typically (49.9%) related to *Difficult situations of life*, often connected to significant personal trauma (e.g. death, divorce, breakup), mental health problems (e.g. depression, insomnia), illness (e.g. cancer), separation from the loved ones (while travelling alone, being abroad for longer periods of time), being especially lonely (e.g. being bullied, disconnected from another person) or having suffered personal failures (e.g. unemployment). 2.6% of the participants did not want to give any details about their experiences, as they felt the topic was too private to be shared, even anonymously. These accounts were removed from the qualitative dataset.

Often the music was sought for *Comfort and emotion regulation* purposes (11%), but people also experienced memorable moments related to sad music in *Unexpected situations* (12.8%), such as hearing music from the radio whilst commuting, or in public venues. On the other hand, the other kinds of experiences were related to *Reminiscing* (11.6%), *Aesthetic experiences* (8.6%), moments of *Introspection or meditation* (6.3%), and *Pleasure* (2.9%). Although listening to sad music was frequently reported to take place home alone, a noteworthy proportion (19.1%) of participants’ accounts described public events, such as concerts or religious events, or even intimate moments of *Social sharing* (5.3%) related to music listening. Also, memorable experiences were linked with moments of *rest or recovery* (3.0%), such as listening to sad music after a long day of work, or in bed before falling to sleep.

**Fig 1:**
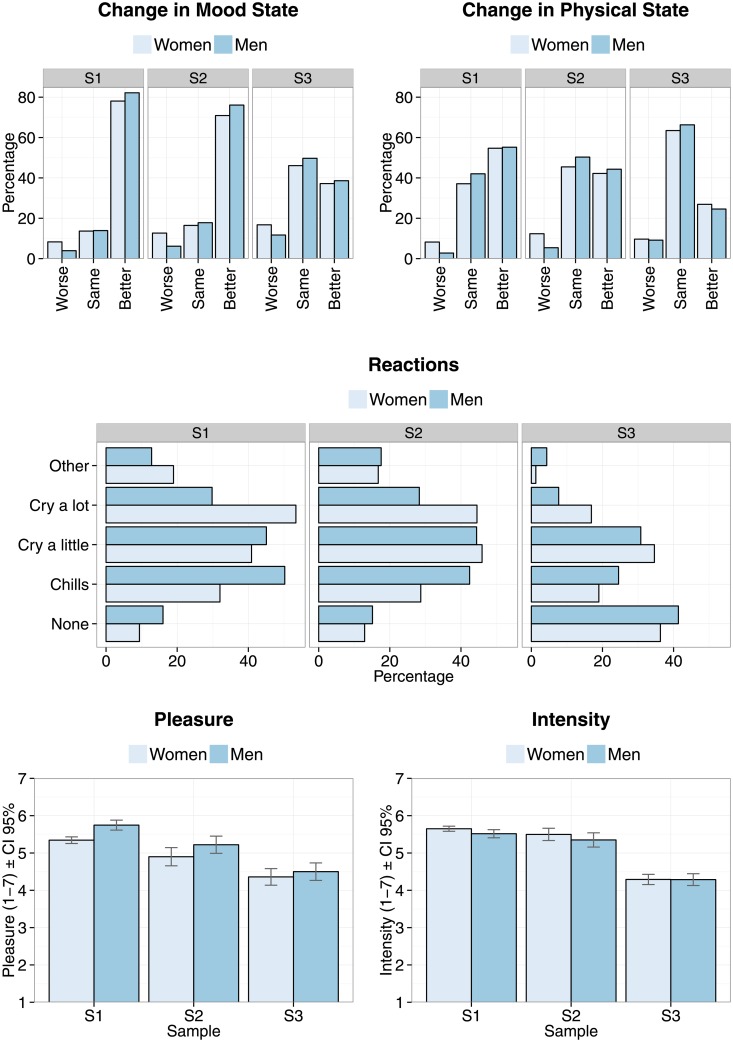
Percentage of participants indicating physical or mental reactions, changes to states, and pleasure and intensity related to memorable sad music episodes across samples and gender.

According to the responses, these experiences had typically occurred more than a year ago (41.9%), involving music chosen by the participants (71.8%) that was extremely familiar (48.7%) or at least very familiar (26.1%) to the respondents. In the situation remembered, the participants listened to sad music for a wide range of durations (from less than 5 minutes, 15.1%, to 5-10 minutes 21.0%, 10-30 minutes 23.6%, 30-60 minutes 19.0%, and more than 60 minutes 20.4%). The exact structure of the emotions experienced whilst listening to sad music in this memorable example are analysed in detail in the next section, but their emotions were typically highly intense (M = 5.56, SD = 1.20 on a scale of 1-7) and pleasurable (5.35, SD = 1.57), although these differed across samples, see Fig 1.

Between 20% and 50% of the participants reported reacting to music physically, mainly by having “chills or goose bumps” (approx. 35% in the S1 and S3, 20% in the S2), by “crying a little (just wet eyes)” (30-45% across the samples), “I cried a lot” (wet eyes and sobbing) (10-50%), or other physical reactions (less than 20% of the time), see Fig 1.

Other physical reactions such as dancing, being immobile, and singing were mentioned in the open comments. When participants were asked to compare how they felt mentally after listening to the music compared to how they felt before, most of the samples indicated that they felt better than before, although again, samples differed in proportion of answers given to each category (see Fig 1 for details). A related question about the changes in physical states painted a similar but more subdued picture, since a only those keen on sad music (S1) reported feeling physically better than before whereas the other samples commonly reported having similar physical state after listening as before the episode. It is also worth highlighting the proportion (11.8% and 11.7% for S2 and S3) of people reporting that their mental state actually worsens after these memorable experiences, suggesting either that these experiences are not entirely pleasant or that they may also lead to rumination [33].

The physical and mental reactions and changes also exhibit strong gender differences; for physical reactions, women report more crying than men in all samples (*χ*^2^>14.1** for S1-S3), which is consistent with crying in general, where women have been shown to be higher in crying propensity and crying frequency [34, 35]. There are also gender, musical expertise, and sample differences across the ratings of intensity and pleasure of the memorable experiences. Samples themselves differ in intensity, *F*(2,1953) = 8.499, *p*<.001, where sad music listeners (S1) report, rather unsurprisingly, higher intensity ratings (M = 5.6, SEM = 0.03) than the other samples (M = 4.29 SEM = 0.05 and M = 5.42, SEM = 0.06 for S2 and S3, respectively). Women generally report higher (M = 5.45) intensity than men (M = 5.20), *F*(1,1953) = 5.4, *p*<.05, but this is only evident in S1. Moreover, musical expertise displays a clear main effect of intensity, where higher expertise yields higher intensity ratings, F(4,1953) = 12.9, p<.001. None of the factors interact, however. The ratings of pleasure display a similar story; significant main effects of sample (*F* = 21.7, *p*<.001), gender (*F* = 15.2, *p*<.001) and musical expertise (*F* = 10.7, *p*<.001) emerged without any significant interactions between them. The ratings of pleasure are highest in S1 (M = 5.5, SEM = 0.03), and lower in the two samples less interested in sad music (M = 4.4, SEM = 0.08 and M = 5.1, SEM = 0.08 for S2 and S3, respectively). Contrary to the gender differences in intensity, ratings of pleasure are actually higher for men than for women (across all samples, men M = 5.3, SEM = 0.06 and women M = 5.1, SEM = 0.04), although this is again driven by the S1 and the comparison within the other samples (S2 and S3) show no gender differences in pleasure.

### Structure of emotions in memorable experiences of sad music

The participants were asked to characterise the emotional quality of their memorable experience using a list of 36 emotion terms. The response frequency of each term across the three samples is shown in Table 2. The top rankings across the samples are held by sadness, being moved, and pleasant melancholia, comfort, and peacefulness, which all have slightly different emphases and emotional meaning. There are noteworthy differences between the UK (S2) and Finnish samples (S1 and S3): The amount of terms indicated differs across the samples, S1 indicated a mean of 8.78, S2 4.7 terms, and S3 7.8 terms. The largest differences are related to nostalgia (3rd most important for the S2, 10th in the other samples) and wonder (rarely—7%—mentioned in the S2, mentioned by 30-38% of the S1 and S3). Whether these discrepancies are due to language and culture or other sample related differences (age, etc.), it is difficult to know at this point. Otherwise, there seems to be a general consensus on the terms and their suitability for describing the emotions induced by memorable sad music experiences.

**Table 2:** Percentage of participants indicating each emotion term in all samples.

S1	S2	S3	Term	S1	S2	S3	Term
76.0	58.4	72.5	Sadness*	19.6	13.5	17.9	Satisfaction
73.2	43.4	68.4	Being moved	19.5	3.1	18.1	Humiliation
48.8	16.9	40.3	Melancholia†	15.2	3.8	12.3	Fear
48.6	16.9	42.5	Comfort	15.1	8.1	13.3	Frustration
41.9	27.6	41.5	Peacefulness*	14.5	10.6	12.1	Power
41.5	13.0	34.1	Sad but elated	14.2	6.3	12.6	Guilt
40.6	12.8	37.9	Powerlessness*	13.2	2.9	8.5	Rapture
37.7	7.0	30.2	Wonder*	12.4	8.3	10.9	Depression
37.5	11.9	32.1	Grief-stricken	9.6	8.5	12.6	Anger
36.8	35.5	33.1	Nostalgia*	7.7	4.9	6.5	Elation
36.1	14.6	35.5	Downhearted†	7.1	9.7	5.6	Uplifted
33.0	15.3	28.3	Relief	6.8	2.5	8.2	Other
27.4	13.3	21.7	Self-pity	5.7	2.9	5.3	Disgust
25.8	25.2	19.8	Tenderness*	5.3	7.0	5.1	Tension*
25.6	13.7	21.0	Joy*	5.0	8.1	4.6	Dismay
25.6	13.0	20.8	Transcendence*	3.3	5.2	3.1	Contempt
22.3	7.4	19.1	Indescribable	2.7	1.6	5.3	Irritation
21.0	6.3	19.8	Anxiety	1.9	10.6	1.4	Don’t know

Table notes. Terms marked with * can be found in the GEMS scale.

^†^ The full terms were “Pleasant melancholia” and “Being downhearted”.

As predicted by past studies [6, 36] using GEMS to assess the emotions relevant in sadness related to music, there is large diversity present in the responses. However, there seems to be pattern where similar terms that refer to negative aspects (e.g. “being grief-stricken”, “being downhearted”, “anxiety”) and positive aspects (e.g. “comfort”, “nostalgia”, “wonder”, “relief”) of the emotional experiences frequently co-occur. In the case of some of terms (“powerlessness”, “being moved”, and “indescribable feelings”), it is difficult to determine whether they are positive or negative in this context. For subsequent analyses, it is also interesting to note that the terms in the GEMS scale (9) are well spread in their frequency of use (these are marked with asterisks in the Table 2), possibly implying a good coverage of the experiences.

To explore the possible structure behind these indicators of emotions in sad experiences linked with music, a factor analysis was applied to the response matrix obtained from S1. Before applying the factor analysis, the terms that were not helpful for the interpretation were removed (“don’t know”, “other”), and those terms that were rarely mentioned (less than 15% of the time, see Table 2) were discarded. The factorability of the ensuing matrix was sufficiently high using polychoric correlations between the columns of the binary data (MSA = 0.82, all items > 0.74, well above the suggested limit of 0.60). Velicer’s MAP analysis was utilised to determine the number of factors extracted, which suggested an optimum of 3 factors that capture the underlying responses in a satisfactory manner (*χ*^2^(133) = 1282.5, *p*<.001, fit = 0.689, RMS = 0.069). To clarify the interpretations of the components across the factors, principal components analysis with promax rotation was used in the final estimation of the loadings. To further prune and improve the model, items with loadings below 0.32 (“humiliation”) and those obtained lower than 0.60 alphas within the factors (“relief”, and “being moved”, “nostalgia”, “indescribable feelings”) were removed, resulting in a slightly better model (*χ*^2^(63) = 1080.2, *p*<.001, fit = 0.753, RMS = 0.081).

**Fig 2:**
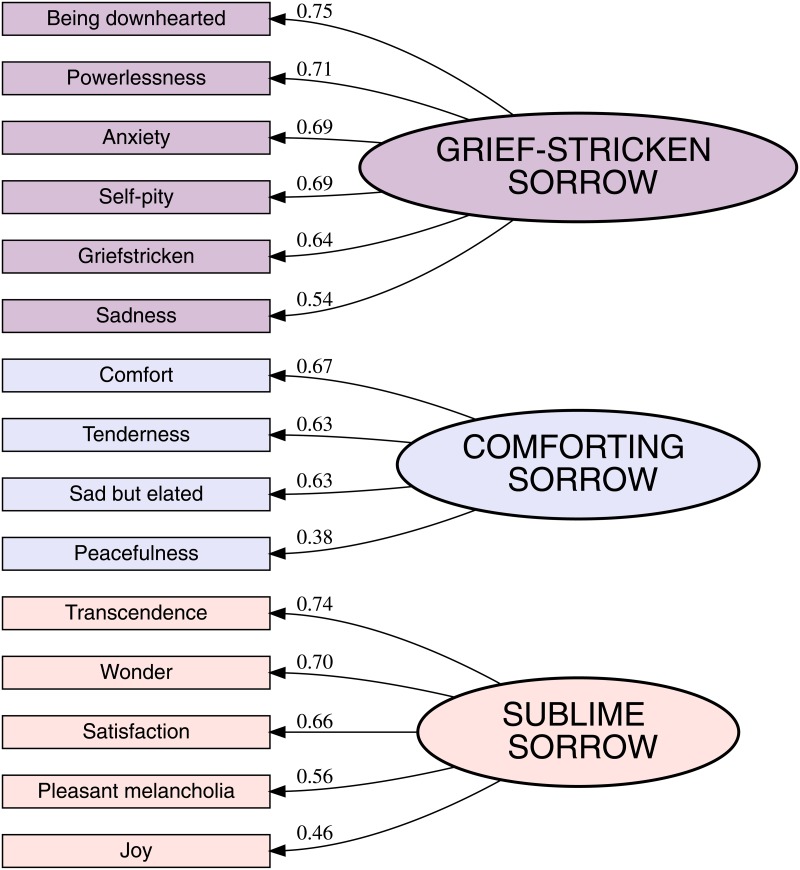
Rotated factors loadings for the three latent affect factors.

The final three-factor model explained 46.0% of variance in the nomination of the emotion terms (PC1 19%, PC2 14%, PC3 13%) and the intra-factor consistencies were high (Cronbach *α*s of 0.87, 0.80, and 0.75, for PCs 1 to 3, respectively). See Fig 2 for the rotated loadings. There is one curious cross-loading, namely the most popular term in the list, “sadness”, which loads both onto first and second factors, suggesting that it can be interpreted to be either a negative (factor 1) or a positive experience (factor 2). Since the survey concerned sadness in association with music, it is no surprise that this term is frequently chosen, but the way it was interpreted can vary considerably. Removing this item would slightly improve the consistency of the model, but sadness is now retained at present.

The three factors can be interpreted to represent “Grief-Stricken Sorrow”, “Sublime Sorrow”, and “Comforting Sorrow” with clearly distinct profiles in terms of the core affect dimensions such as valence and arousal. The first factor, labelled as *Grief-Stricken Sorrow*, has all the negatively valenced terms. Factor 2, titled *Sublime Sorrow*, is the most positive, containing “joy”, “transcendence”, and “wonder” and factor 3 titled *Comforting Sorrow* is also positively valenced but a low arousal variant with items such “tenderness”, “comfort”, and “sad but elated”. An additional validation of this simple core affect interpretation of the factor solution can be achieved by comparing the terms with the valence, arousal, and dominance ratings of the same affect terms [37]. In such an analysis, the factor loadings of the first factor correlate negatively with valence (*r*(15) = -.80, *p*<.001), the third factor negatively with arousal (*r*(15) = -.43, *p* = .078), and the second factor loadings are positively correlated with dominance ratings of the terms (*r*(15) = -.518, *p*<.05).

To understand the factor structure better, the factor correlations were checked for consistency across the datasets. This three-factor model was also applied to the other samples (S2 and S3). This revealed that the factors are not perfectly orthogonal since oblique rotation (promax) was utilised; however, the correlations are in general small and consistent across the datasets. The first factor (*Grief-Stricken Sorrow*) is negatively correlated with the *Comforting Sorrow* (*r* of -.11, -.08, and .19 for S1, S2, and S3, respectively), whereas *Sublime Sorrow* exhibits slightly higher negative correlations (*r* of -.36, -.37, and -.17 for S1, S2, and S3, respectively). *Comforting* and *Sublime Sorrow* do also show positive albeit small correlations with each other (*r* of .31, .27, and .23 for S1, S2, and S3, respectively).

### EFA with several sadness-specific and theory-driven models

The three-factor solution shown Fig 2 is perhaps the optimal one for this dataset; nevertheless, it is prudent to formulate alternative structures to explore how much variance is being explained either by removing or adding factors, or by comparing these sadness-specific models to other theory-driven models such as GEMS or affective circumplex model.

For the purposes of the comparisons, the data containing the frequent terms was analysed using structural equation models (SEM).). This was achieved by constructing 1 to 4 factor variants of the sadness-specific models from the terms, in addition to several theoretically-driven models such as the GEMS and affective circumplex models. Since the structural equation fit indices are sensitive to model parsinomy [38], an attempt was made to keep the models comparable with respect to the number of terms. Since our implementation of the main contender, GEMS, has nine terms, all other models were also applied using nine terms. The terms were chosen by selecting those that were most reliable within each factor of the model according to interrater agreements (Cronbach *α*s).

Two pruned, sadness-specific GEMS models were created. These were based on an optimal reduction of the nine terms in the GEMS model either to two or three factors based on the principal component analysis with the present data. A three-dimensional, sadness-specific GEMS had “feeling moved”, “wonder” and “transcendence” in Factor 1, “sadness”, “powerlessness”, and “tension” in Factor 2, and “joy”, “tenderness”, and “nostalgia” in the Factor 3. This is akin, but not identical, to the second-order factors in [21], in which the UNEASE factor consists of “tension” and “sadness” factors (similar to the Factor 1), VITALITY contains “power” and “joyful activity”, and SUBMILITY comprises “tenderness”, “nostalgia”, “peacefulness”, “transdence” and “wonder”, which form new combinations of structures obtained in the principal component analysis of the sample S1. A two-factor version of the GEMS was similarly implemented, by forcing the nine terms into two factors (Factor 1 consisted of “joy”, “being moved”, “wonder”, “transcendence”, “tenderness”, and “nostalgia” and Factor 2 contained “sadness”, “powerlessness”, and “tension”).

Four theoretically inspired models were added to comparisons to critically assess the goodness of the sadness-specific models. First, we adopted the original formulation of the GEMS model by representing the nine emotions with three second-order factors [21]. Models relating to affective circumplex [39] were created by first associating all the 36 emotion terms with the normative ratings of valence and arousal for these terms [37]. Then a two-factor valence model was formed by dividing terms into positive and negative designations according to mean valence values and taking the nine most reliable ones (highest Cronbach *α*s within the factors). This model had “joy”, “wonder”, “transcendence”, “rapture”, “peacefulness” as positively valenced terms (factor 1) and “powerlessness”, “self-pity”, “anxiety”, and “being downhearted” as the negatively valenced terms (factor 2). In a similar fashion, a two-factor arousal model was created, which consisted of the most consistently used high arousal terms (“anxiety”, “indescribable”, “powerlessness”, “anger”) and low arousal terms (“sad but elated”, “comfort”, “pleasant melancholia”, “tenderness”, and “relief”). All the models thus postulated are comparable in terms of model parsimony, since they have from 2-4 factors represented by 9 items, yielding comparable though not identical degrees of freedom in the model evaluations.

The models described were used to predict the evaluations given by the S1. The results concerning the four sadness-specific models, displayed in the upper part of the Table 3, are—as expected—consistent with the first analysis of the optimal structure for this dataset, showing highest fit for the three-factor solution. The model fit (RMSEA) for 2 to 4 factor models are decent, although the incremental fit index (CFI) suffers from overspecification since the models have 4-7 items that overlap considerably within the factors. For this reason, the models pruned using the within-factor alpha trimming principle (retaining highest alphas), as explained above, provide a more sensible point of model comparison.

**Table 3:** The fit indices of the EFA models of emotions with sad music based on S1 (*n* = 1577).

Model	*χ*^2^	*df*	CFI	RMSEA	RMSEA CI_90_
*Sadness-specific*					
One-factor	2423	152	0.543	0.097	0.094-0.101
Two-factor	1366	151	0.755	0.071	0.068-0.075
Three-factor	1280	149	0.772	0.069	0.066-0.074
Four-factor	1422	146	0.743	0.075	0.071-0.078
*Sadness-specific Pruned*					
Three-Factor Pruned	124	24	0.940	0.051	0.043-0.061
Two-factor GEMS terms	213	26	0.819	0.068	0.059-0.076
Three-factor GEMS terms	145	24	0.884	0.057	0.048-0.066
*Theoretical*					
Three-factor GEMS	327	24	0.717	0.090	0.081-0.098
Two-factor Valence	219	26	0.906	0.069	0.060-0.077
Two-factor Arousal	171	26	0.881	0.060	0.051-0.068

Of the three pruned models—the best model from the sadness-specific analysis (three-factor sadness model) and the two sadness-specific GEMS models—the pruned three-factor model reaches acceptable fit in terms of both RMSEA and CFI indices [38]. It is also significantly better than the best unpruned sadness-specific model from the previous analysis (*χ*^2^ difference = 1156, *p*<.001). The two-factor models using the GEMS terms optimised with this data fail to reach acceptable fit (CFI<.85), whereas the three-factor model with the GEMS terms fares better by obtaining a marginal goodness-of-fit (CFI = .884) and acceptable error rate (RMSEA < .057). Both GEMS models are, however, significantly worse than the three-factor sadness-specific model (*χ*^2^ difference = 89.4 and 20.7, both *p*<.001).

The three theoretical models, which are not specific to sadness except by choice of terms from within the 36 term vocabulary, produce a spread of fit with the data. The theoretical formulation of GEMS with three factors fails to capture the correlation matrix of the responses (CFI<.85 and marginal RMSEA value, see Table 3). This is perhaps to be expected, considering that the range of emotional experiences differs to that which the original instrument was designed to capture. The two-factor valence model reaches acceptable fit (CFI>.90 and RMSEA<.08), suggesting that simply dividing the terms into negative and positive designations allows the capture of an essential aspect of sad experiences, although the three-factor sadness-specific model provides significantly better fit. The two-factor arousal model, however, is not appropriate for explaining the underlying correlation matrix of emotion terms, since it fails in the incremental index (CFI<.90), although the RMSEA values are acceptable.

To summarise, two different types of models seem to account for the responses given on relevant emotions in memorable experiences, one derived from the data itself (three-factor sadness-specific model) and the other from the affective circumplex (the two-factor valence model). The models derived from GEMS terms seem to have an insufficient number of negative terms that are needed to account for the kinds of experiences often encountered in these situations. A model constructed with arousal also fails to capture the essential aspects of the experiences. However, these exploratory factor analyses can only provide ideas and materials for a more rigorous comparison, best carried out with separate data and CFA.

### CFA with the best models from EFA

From the EFA analysis stage, three plausible models were taken into CFA: the best sadness-specific model (the three-factor pruned model), the best affective circumplex model (the two-factor valence model), and the best GEMS model (three-factor GEMS terms model). These models were applied to the two unused datasets, to the S2 and S3. The purpose was to ascertain which models retain the predictive capacity to new datasets and how well they generalise across the materials that have been collected in two different countries. The CFA analyses were carried out with Lavaan (0.5-18) [40] of R [41]. Table 4 summarises the model performances across the two new samples.

**Table 4:** The fit indices of the CFA models from S1 applied to S2 (*n* = 445) and S3 (*n* = 414).

Model	*χ*^2^	*Df*	CFI	RMSEA	RMSEA CI_90_
*Sadness-specific*					
S2	40.6	24	0.918	0.039	0.016-0.060
S3	51.8	24	0.925	0.053	0.033-0.073
*Valence*					
S2	56.9	26	0.889	0.052	0.033-0.070
S3	71.9	26	0.896	0.065	0.048-0.084
*GEMS*					
S2	126	24	0.615	0.098	0.081-0.120
S3	55.1	24	0.874	0.056	0.037-0.076

The sadness-specific model appears to have an acceptable fit to the new datasets, considering both incremental and error indices (CFI >.90 and RMSEA<0.08), whereas the valence and the GEMS models fail at least in one of the fit measures (all CFI are <.90). Looking at the results more closely, the sadness-specific model is more successful in explaining the responses from the S2 than the S3. This is surprising, considering that the model was developed with the S1 consisting of Finnish participants, which might be assumed to more closely approximate the S3, which was a quota sample from the same country. This best model does not appear to suffer significantly from applying it to either of the new samples, since the fit indices are nearly at the same level as those in the first evaluation of the model with sample S1 (ΔCFI is -0.0185 and ΔRMSEA is +0.005 between EFA and the average of two datasets in the CFA).

The valence model outperforms the GEMS model in the both new datasets and also suffers little loss from EFA to CFA (ΔCFI = -0.0135 and ΔRMSEA = +0.011). The GEMS model is particularly weak for the S2 and is visibly impaired when the model is taken to the new datasets (ΔCFI = -0.1395 and ΔRMSEA = -0.02). Even though the GEMS model was optimised with sadness in mind in the EFA stage, relying on a compact pool of terms seems to constrain the model in an unfavourable fashion in this context. Switching the language between the datasets (Finnish and English) is unlikely to be the sole reason for the failure of this model since the most effective models (the sadness-specific and the valence model) do predict the responses across the countries in a reliable fashion.

Having established that the three-factor sadness-specific model is adequate in explaining the range of affective responses in the available datasets, the influence of demographics on the model factors is explored next.

### Individual differences in the three factors of sadness

To explore whether the structure of sadness is similar across participants in all samples, the scores representing the three components from the best model (three-factor sadness-specific model using the original, 15-item formulation shown in Fig 2) were subjected to ANOVAs across the participants’ gender, age and musical expertise. Broadly summarising the results (displayed in Table 5), the S1 displayed the largest differences for all factors, the S2 had no significant main effects except gender for the first component, and the S3 exhibited mainly gender effects. Looking at the factors separately, Factor 1 (*Grief-Stricken Sorrow*) showed significant main effects of Age (*F*(5,1524) = 20.8, *p*<.001) and Gender (*F*(1,1524) = 12.7, *p*<.001) but not Musical expertise (*F*(4,1524) = 1.1, *p* = .37) in the S1. Young participants obtained higher scores (highly significant negative linear contrast for age, *t* = 8.5, *p*<.001) and women scored higher (M = 0.051, SEM = 0.029) than men (M = -0.147, SEM = 0.052), suggesting that these negative feelings linked with episodes of sad music are more prevalent in younger people and women. This is also true for the S3 concerning gender (*F*(1,366) = 19.3, *p*<.001), where women again scored higher (M = 0.11, SEM = 0.067) than men (M = -0.30, SEM = 0.062), and the S2 obtained a similar gender difference (*F*(1,432) = 8.2, *p*<.01) in this factor. Again, this is line with the past research showing women reacting more strongly to negative emotions than men [42].

**Table 5:** ANOVA results of the background differences for sadness components across the samples.

Factor and Sample	Gender	Age	Expertise	Interactions
*Grief-Stricken Sorrow*				
S1	12.7***	20.8***	1.1	-
S3	19.3***	1.6	0.9	-
S2	8.2**	0.1	-	-
*Sublime Sorrow*				
S1	2.3	1.1	5.8***	
S3	1.9	2.2	4.4**	G x E*
S2	1.0	1.6	-	
*Comforting Sorrow*				
S1	98.4***	3.0**	5.4***	-
S3	12.6***	1.4	2.2	A x E*
S2	3.3	1.6	-	

Table notes.

* *p* <.05,

** *p* <.01,

*** *p* <.001.

Interactions refer to the first letter in ANOVA factors (G = Gender, A = Age, and E = Expertise).

Factor 2, which captures the feelings of transcendence and joy (labelled as *Sublime Sorrow*), exhibited only significant main effect of Expertise for the S1 (*F*(4,1524) = 5.8, *p*<.001) and the S3 (*F*(4,1524) = 4.4, *p*<.01). The more musical expertise the participants possessed, the higher the scores for this factor (fitted with highly significant linear contrast, t = 3.5 and 2.7, *p*<.01, for S1 and S3, respectively). There was also an interaction between Gender and Age in the S3 (*F*(4,366) = 2.7, *p*<.05) which mainly highlights that Finnish men over 65 years obtain particularly low scores in *Sublime Sorrow* (M = -.77, SEM = .25 whereas the mean for the similar aged women is.11, SEM = .38).

For the third component (*Comforting Sorrow*), S1 exhibited significant main effects of Gender (*F*(1,1524) = 98.4, *p*<.001), Age (*F*(5,1524) = 3.0, *p*<.01) and Musical expertise (*F*(4,1524) = 5.4, *p*<.01). Women obtain lower scores (M = -0.14, SEM = 0.027) than men (M = 0.40, SEM = 0.052) and age shows differences in the older year categories (highest scores in the 65-year and older participants). Musical training was positively related to factor scores (*t* = 5.3, *p*<.001 with a linear contrast). This suggests that the experiences of tenderness and comfort in relation to sad music may be more common among musically trained people than amongst those with less training, at least in those who do prefer to listen to sad music (S1). The S3 did show a main effect of Gender similar to that of the S1 (*F*(1,366) = 12.6, *p*<.001) where again women display lower scores (M = -0.31, SEM = 0.061) than men (M = 0.00, SEM = 0.068). Everything considered, there are significant differences in emotions experienced that relate to simple demographic variables, although the relevance of these differences observed in large samples remains to be explored in future.

Gender displays the most differences in the analysis, which is in line with other studies of emotions induced by music. For instance, women tend to favour different regulation strategies related to negative emotions than men [1], score higher for contagion as a principle for accounting negative emotions [6], and generally use music more for mood regulation [43]. Such patterns do not seem to be specific to music, since gender differences in self-reports of emotion has been observed repeatedly across the affective sciences [44], although neural indicators seem to underplay such differences [45]. The key component here is assumed to be empathy, which is multidimensional ability to understand emotional states of the others. Although empathy needs to be divided into different subcomponents, such as cognitive or affective [46] and their exact nature is still undefined, empathy seems to be consistently different across gender [47]. The present study cannot pursue the putative explanations, but at least the gender roles and emotion recognition advantage by women [48] are consistent with the differences observed.

### Prevalence of broad emotion types related to musical sadness

In the final question, we asked respondents to answer “how often have you experienced the following feelings in the context of sad music”, and offered them the three broad categories of emotions relevant for sadness and music that were obtained from the previous study [18]. This question was phrased to be independent of the memorable experience and addressed the issue of prevalence.

The emotion categories were explained with several labels. The category titled *Sweet Sorrow* was expressed with “feelings of comfort”, “relief”, or “joy”. *Melancholia* consisted of “depression”, “gloom”, and “sadness” and *Grief* was defined as “feelings of deep hatred”, “grief”, or “loss”. Whilst these three emotion categories were not identical to the three factors identified in the analysis of structure of sadness in the memorable experiences, they bear strong similarity to them. The first one, *Sweet Sorrow* is similar to *Comforting Sorrow* (Factor 2) although the term joy itself is here associated with *Sublime Sorrow*. *Melancholic* sadness bears closest similarity with *Grief-Stricken Sorrow* since the terms (“depression”, “gloom”, and “sadness”) correlate best with this factor. The third category, *Grief*, of course, relates to Factor 1 labelled *Grief-Stricken Sorrow*. Thus, the factors identified in the present analysis classify sadness-related experiences into two different types of pleasure and one more aversive group of emotional experiences, whereas the previous analysis illustrated more the different qualities of negative experiences [18].

**Fig 3:**
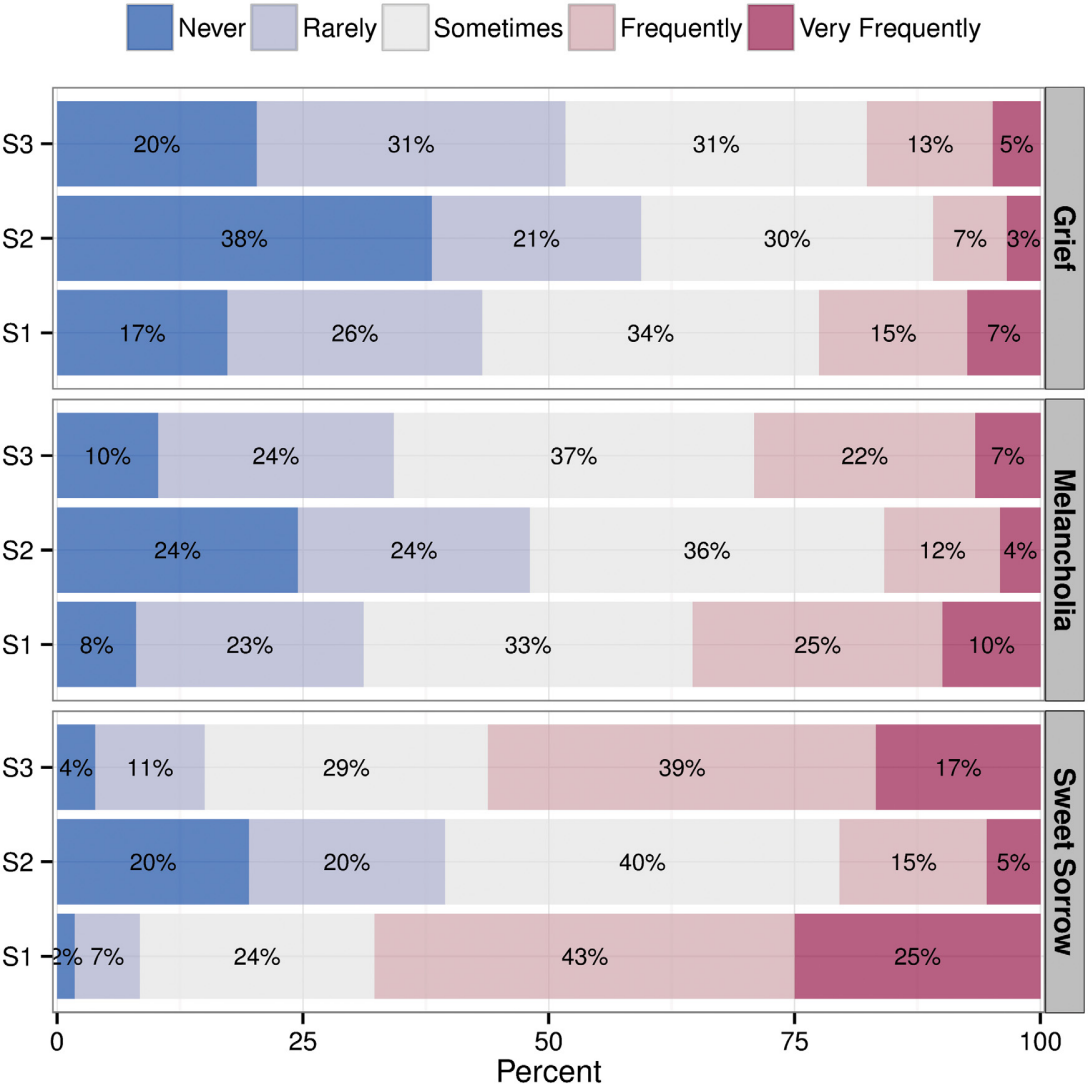
Frequency of responses to three broad types of emotions involved in music and sadness across the samples.

Although we can observe the frequencies of the emotion terms relevant for memorable experiences in Table 2, the summary question provides a way of validating the relevance of different types of emotions in the context of music in general. Fig 3 summarises the proportion of responses to each of the three emotion categories for all samples. The majority has frequently experienced *Sweet sorrow* (“frequently” or “very frequently”, 68%, 20%, and 57% for S1, S2 and S3, respectively), whereas the Melancholic variety seems to be rarer (35%, 16%, and 29% of the samples S1, S2, and S3 answered “frequently” or “very frequently”) though not uncommon. The deeply negative feelings of sadness, involving feelings of hatred, grief and loss, are the least common emotional experiences in the context of music. 21% of the S1, 10% of the S2, and 18% of the S3 answered “frequently” or “very frequently” to this question, which is also consistent with the frequency of similar terms in the memorable experiences (being grief-stricken 36.3%, anger 10.4%, and depression 12.0%, see Table 2). Although these emotions form the least common broad category of experiences in the context of music and sadness, a fifth of participants reporting that they frequently encounter these emotions still renders this an important class of experiences.

### Characteristics of three types of sadness experiences

To understand whether the three identified types of emotions in memorable sadness experiences are distinct from each other, and how they are connected to the reasons, mechanisms and reactions, these components were subjected to regression analysis. To minimise the overfit, the largest sample (S1) was used in building a lasso regression model where 50% of the observations were used in the training phase with 10-fold cross-validation and the remainder of the data used for testing. The lasso was chosen to eliminate redundant variables [49] and the evaluation scheme provided a robust measure to identify which combinations of variables would explain the three sadness scores of each participant. In order to probe the model developed and assessed with the S1, the model was also used to predict the scores of the samples S2 and S3.

As indicated by Table 6 in which the regression analyses are summarised, the three types of sadness experiences display reasonable prediction rates (*R*^2*adj*^ 0.24 to 0.44) with the 8 to 10 predictors. *Grief-Stricken Sorrow* is associated with reflective reasons, high intensity and physical reactions, and lack of pleasure and changes in mental state (negative coefficients in the latter two). Given the free responses to these experiences which highlighted their links with funerals and exceptional situations of coping with loss, the pattern of significant coefficients matches these and accentuates the negative emotional impact of these experiences. *Comforting Sorrow* is linked with the mechanisms of beauty and memory, with belonging as the reason; these experiences are highly pleasurable and lead to positive mental and physical changes. Again, the coefficients are connected to the contents of the experiences obtained in open responses; such experiences often relate to rest, meditation, and introspection. Finally, experiences of *Sublime Sorrow* are characterised by beauty, aspects of the music itself, relaxation, pleasure, and positive changes in mental state. Six of the significant features for *Sublime Sorrow* overlap with *Comforting Sorrow* with the main exceptions being the music as a mechanism for these experiences, and the fact that reflection and intensity operate in the opposite way in *Sublime sorrow* when compared to *Comforting Sorrow*. The experiences of *Sublime sorrow* are strongly characterised by beauty and pleasure, and linked with the actual music.

**Table 6:** Standardised regression coefficients to the Three Emotion Types across Mechanisms, Reasons, and Reactions to memorable sad music experiences (S1, *n* = 1577).

	Grief	Comforting	Sublime
Mechanism—Beauty		0.139	0.197
Mechanism—Memory		0.111	0.121
Mechanism—Music			0.202
Reason—Reflection	0.454	0.018	-0.222
Reason—Relaxation	-0.152	0.072	0.202
Reason—Belonging	0.015	0.106	
Intensity	0.185	0.032	-0.057
Pleasure	-0.187	0.191	0.234
Physical change	-0.106	0.077	0.051
Mental change	-0.199	0.114	0.115
Physical reactions	0.160		0.022
**Model fit**	*R^2adj.^*	*R^2adj.^*	*R^2adj.^*
	0.438	0.238	0.371
**Model application**			
S2	0.237	0.295	0.277
S3	0.384	0.250	0.346

Table notes. Lasso regression with *λ* = 0.1 and 50% sample split for training and testing with 10-fold cross-validation.

When these models are applied to the other two samples (S2 and S3), they generalise fairly well. For *Grief-Stricken Sorrow*, there is a 20% decrease when applied to the S2 and 5% dip in the variance explained when the model is applied to the sample S3. For *Comforting Sorrow*, the model applied to the new samples shown an increase (+6% and +1%) in prediction rates. In the case of *Sublime Sorrow*, there is a decrease in the fit (-9% and -2%, for S2 and S3 samples).

When other relevant variables—such as Attitudes to Sadness in Music (six factor scores)[22]—are inserted to the regression models developed with the sample S1 and shown in Table 6, a small amount of additional variance can be explained (+4% for *Grief-Stricken Sorrow*, 0% for *Comforting Sorrow*, and +1% for *Sublime Sorrow*). When the question of whether the participants had experienced grief, melancholia or sweet sorrow is added to the models, the variance explained for each factor show substantial improvements (+9% for *Grief-Stricken Sorrow*, +2% for *Comforting Sorrow*, +2% for *Sublime Sorrow*). These improvements also carry over to predicting the factor scores in other datasets, improving their fit (*R*^2*adj*^ increases from 0.01 to 0.06). It suffices here to conclude that the main reasons, mechanisms and reactions to these memorable experiences explain the underlying emotional experiences, and prior experiences and attitudes may be particularly relevant in explaining the experiences of Grief, where the most significant improvements were observed.

The final analyses examined the extent of differences between the three sadness factors in terms of the underlying descriptors. A linear discriminant analysis of the factor scores converted into categories by taking the maximum score for each participant addresses this question. This analysis was carried out with a partitioning and cross-validation scheme similar to that of the regression analysis and the number of observations for class was fairly balanced (39%, 31%, and 30%, for *Grief-Stricken Sorrow*, *Comforting Sorrow* and *Sublime Sorrow* factors, respectively). The overall classification accuracy of the three factors using multiclass ROC [50] was 0.781 (Wilks *λ* (2,22) = 0.62, *p*<.001), where the *Grief-Stricken Sorrow* is best predicted (78.3% classification accuracy on the test set), *Comforting Sorrow* (65.4%), and *Sublime Sorrow* (71.3%). With the baseline rate at 38%, these are good prediction rates and driven by few variables such as Pleasure (ROC = 0.73, 0.69, and 0.73 for three factors) and Reason—Reflection (ROC = 0.69, 0.64, and 0.69 for *Grief-Stricken Sorrow*, *Comforting Sorrow*, and *Sublime Sorrow*, respectively). Again, this discriminant model also delivers above chance level prediction when applied to the other samples; an equal rate of classification for sample S3 (ROC = 0.784, Wilks *λ* = 0.56, *p*<.001), and a slightly lower rate for S2 (ROC = 0.680, Wilks *λ* = 0.72, *p*<.001). In summary, the succesful discriminant analysis of the factors based on the underlying reactions, reasons and mechanisms defends their role as independent, separate experiences.

## Discussion

Reactions to memorable experiences of sad music were studied by means of a large-scale survey that explored the reasons, mechanisms, and emotions of such experiences. Memorable experiences linked with sad music typically occurred in relation to extremely familiar music, that caused intense and pleasurable experiences, which were accompanied by physiological effects (such as moist eyes, chills, and tears) and positive mood changes in about third of the participants.

A consistent set of reasons for these experiences was identified, one relating to relaxation, another to reflective processes, and the final one to belonging, findings that bear similarities to previous accounts of reasons for engaging with sad music [6, 18, 19]. However, findings related to the discovery of a finer structure of emotional experiences within this broad category of sadness diverge somewhat from those of previous accounts, and so will now be discussed at length.

### Structure and prevalence of sadness associated with music

The results identified a consistent structure of three types of sadness experiences relevant to memorable events associated with music-related sadness. The structure discovered was carefully validated across three samples and contrasted with pertinent current models accounting for the same experiences. Each of the factors identified—*Grief-Stricken Sorrow*, *Comforting Sorrow*, and *Sublime Sorrow*—has a distinct profile in terms of the underlying reasons, causal mechanisms and reactions elicited. None of the alternative accounts such as GEMS [21] and valence and arousal framework prevailed in the comparisons with the simplified three-factor model of sadness. Moreover, the discovered structure is congruous with qualitative accounts of sadness induced by music [18] and also bears some similarities with other descriptive frameworks used to explain the reasons for listening to sad music [19].

In a previous study of music associated sadness [18], Joyful sadness experiences were reported frequently (61-92%) by all the samples, whereas the genuinely negative experiences (feelings of grief, deep hatred, or loss) were less frequent, but not uncommon in any of the samples (40-56% of participants had sometimes experienced this type of emotion). The former observation is perhaps not surprising in the context of music and fiction in general, which is inherently pleasant, voluntary, and does not have any direct real-life consequences. The latter observation of genuinely negative experiences is unforeseen, at least with respect to the extant research literature, which is devoid of such descriptions. The true prevalence of the different types of emotions is of course difficult to estimate reliably using retrospective surveys, but this is the first time such estimations have been offered with representative and large sample and carefully constructed questions. Experience Sampling Methods would be needed to establish a more accurate account of the everyday prevalence of such emotions [51, 52].

### Defusing the paradox of pleasurable sadness

The results help us to contextualise several of the conflicting results obtained in the previous studies of music and sadness. First of all, the paradox of pleasurable sadnesss is actually less puzzling if one acknowledges that there are different types of “sadnesses”. Past empirical studies have put particular emphasis on the pleasurable experiences induced by sad music [5, 6, 9, 53], which could be close to *Sublime Sorrow* or at least *Comforting Sorrow* identified in the present study. Only few empirical studies have acknowledged the fact that the experiences induced by sad music might actually be genuinely negative, harrowing and unpleasant [18, 22] although such experiences are acknowledged by ethnomusicological field studies [15, 54]. The third factor in this study (*Grief-Stricken Sorrow*) seems to portray such affective experiences, wherein the thematic analysis of the experiences’ content revealed themes of bereavement, mourning, and loss.

Secondly, there seems to be a clear difference between an aesthetic emotion, such as the one labelled here as *Sublime Sorrow*, and the other type of positive experience, *Comforting Sorrow*. The latter is related to other people, to social relationships, and is typically more reflective than the experiences induced by moving music, which is one of the hallmarks of *Sublime Sorrow*. When looking at the qualitative data, lyrics seemed to play a crucial role within the experiences relating to difficult situations of life. This observation stands out in interesting light when comparing it to a finding by Brattico and her colleagues [12], who discovered that lyrics (or vocal information in general) may be crucial for defining the sadness of a musical piece. Furthermore, in their study, participants judged instrumental sad music as more pleasant and beautiful than music containing lyrics. Thus, it might be that *Sublime Sorrow* is more relevant for the experiences of listening to instrumental music, whereas *Grief-Stricken Sorrow* and *Comforting Sorrow* are induced by vocal music with meaningful lyrics. However, since our participants did not consistently identify the type of music they were describing, this connection can only be speculated.

If the differences related to sadness are unacknowledged, this could lead research to either focus on mood regulation strategies [8] if the emphasis is placed on experiences related to *Comforting Sorrow*, or to aesthetic experiences [55] when the *Sublime Sorrow* is the actual object of study. In some cases, both types of experiences seem to be combined [6], but such strategies need more refined treatment of the experiences involved in sadness before they will lead to genuinely novel insights on the topic.

### Sadness as a strong experience

Due to the way the research question was formulated around a memorable example, these experiences—judging by the high intensity of the experiences, open responses, and prevalence of physical reactions—were closer to Strong Experiences with Music (SEM) than the typical results os music and sadness offered by laboratory-based studies. The SEM experiences, which have been extensively documented by Gabrielsson [56, 57], are collected by asking people to describe their strong and memorable experiences involving music, which is nearly identical to the somewhat narrower question posed in the present study. It is no wonder that there are strong similarities between the SEMs and the present findings; typically more than a year had passed since both SEMs (about 50%) and memorable sad experiences (42%), and the music is familiar or very familiar to the listeners in both cases (54% in SEMs, 74.8% in sad experiences). Also, strong experiences with music often feature a similar list of common physical reactions (crying 24%, chills 10%, and other reactions such as a lump in the throat etc.) observed in the context of memorable sadness experiences.

Probably the most interesting parallel between SEMS and the memorable sadness experiences with music is that truly negative experiences are not that uncommon in SEMs (23%) ([56], p.387). This mirrors the frequency of responses to experiences of truly negative emotions (*Grief-Stricken Sorrow*, rated to be frequent or very frequent by 10-22% of the participants in the present study, see Fig 3). Although space prohibits a more detailed comparison of SEMs and memorable sad experiences, the similarities suggest that memorable sad experiences could be fruitfully interpreted as SEMs, and a closer analysis of the open responses in the framework provided by Gabrielsson could be a useful way to explore the qualities of these special experiences in more detail.

### Methodological implications

The findings of the present study have implications for the content of self-report measures commonly employed to measure emotions associated with sad music. The affective circumplex model does not capture the nuances of these experiences in sufficient detail, which has already been observed in the past studies [6, 9, 36]. Interestingly, Kawakami [5] proposed four emotional factors that are induced by sad music, although unfortunately this was a modest exploratory model since it was based on responses from 44 participants after listening to three nominally sad excerpts of classical music. As such, their study may be considered to have a narrow focus and insufficient quantity of observations for a reliable structure discovery. At the other extreme, the most sophisticated self-report instrument to measure music-induced emotions to date, GEMS [21], seems to lack sufficient power to distinguish the specialised emotions involved in sadness. GEMS model consists of nine separate dimensions of emotions, and has been properly validated with large samples. It has been utilised by several scholars studying experiences induced by sad music [6, 9, 36], but as it is designed to capture a broad range of experiences induced by music, the present study suggests that sadness seems to be a theme that is too specific to approach using GEMS; nevertheless, it still captures a fair amount of the different emotional experiences associated with this specialised topic.

### Limitations and directions for future research

There are several caveats of the present study that need to be acknowledged. First, the findings apply to memorable examples using self-reports, both of which elements have shortcomings. Nevertheless, self-reports are a cost-effective, robust and valid way of capturing emotions [58], even though the underlying mechanisms and reasons might be less accessible to the participants [51]. Also, episodic examples have been shown to be less biased than generic semantic memories [20]. One problem lies in the highly personal nature of music-induced emotions and researcher-chosen music is known to be less effective than any personal selections with autobiographical memories associated with the music [7, 10]. For this reason, the emphasis on memorable experiences provides direct, personal insights into the topic in a way that avoids the need to present researcher-chosen music in an artificial situation to the participants. At the same time, it needs to be acknowledged that it is an open question as to whether similar pattern of emotions are evident in unfamiliar, sad-sounding music [36]. Early findings from laboratory-based listening studies seem to suggest that emotional experiences induced by researcher-chosen music could be best captured by specific sadness-related emotional structure [59], but this requires for further studies utilising a wider range of measures (e.g. psychophysiology and action tendencies).

Another caveat is the selection and the size of the samples. Here two main ways of sampling, convenience (S1) and representative (S2), were utilised to provide alternative perspectives on the topic. Since the samples were from different countries, no direct effects of the sampling method itself could be studied since the sampling technique was confounded with the sampling country. Despite being unable to study the effects sampling technique directly, there was an advantage in having two countries represented in the study. Also, the third sample (S3) was obtained to explore the age and musical expertise differences inherent in the two samples, which brought additional information about the role of these factors to the results.

It must be noted that all past studies [5, 6, 36, 60] involving music and sadness have utilised convenience samples and been significantly smaller in size. Only one broad music and emotion survey has been carried out with a representative sample [1], in which the focus was not on the present topic. Nevertheless, it would be valuable to replicate the survey in a few other Western countries to assess the variability of the results and to obtain better estimations of the prevalence of the different types of sadness experiences associated with music. An even more fascinating issue would be to transport the relevant parts of the survey to a few non-Western countries to learn whether there are any decisive cultural differences within this topic.

The significant individual variation in memorable experiences linked with sad music is an interesting aspect of the findings. Although past studies have either downplayed the importance of individual differences [61] or emphasised their role [1, 36], it was clear that here there was an interaction between the experiences and musical expertise, age and gender, and that differences were more pronounced in samples with higher interest in music. Also, younger and musical trained participants reported stronger emotional responses in general, which is similar to more general trends in emotional reactions across the life span [62]. Unfortunately the present research could not connect the reasons, mechanisms and experiences to personality traits, which seems to be particularly relevant for sadness induced by music [4, 9]. This is, however, easy to remedy in future studies once the right structures and reasons for these emotional experiences have been identified.

### Theoretical implications

There have been several attempts of explaining the enjoyment of sadness induced by music and other fiction. Most of these emphasise the beauty and the lack of real-life repercussions [24, 63, 64], and in light of our findings, only pertain to one kind of emotional experiences (*Sweet sorrow*), but more elaborate theories postulate a more specific mechanism at play. Some assume [8] that whilst sad music and associated memories are painful, these might turn into positive emotions, such as nostalgia, afterwards. Whether such transformations could be characterised in terms of distraction or reappraisal processes [65], remains to be explored in follow-up studies. It is unlikely that this could be classic explanation often dubbed as catharsis, since such strategy seems to be inefficient coping strategy after induced sadness [66]. A more plausible explanation is our inherent need to experience different kinds of emotions, which fiction effectively generates [67]. Finally, going back to functional explanation and biology, Huron has offered a tantalizing explanation, which utilises the ability of fiction to emulate our experience of real sadness, triggering an endocrine response (prolactin) designed to alleviate mental pain associated with significant loss [29]. This hormonal response is experienced as consoling, even enjoyable, when the real-life consequences of the loss are absent. Indeed, early evidence exists that prolactin levels are modulated by positive and negative emotion induction, at least in women [68], but the theory is without corroboration yet. The present findings would suggest that experiences related to *Comforting Sorrow* would be most relevant experiences explained by this theory, whereas the experiences of *Grief-Stricken Sorrow* might still be relevant for the production of prolactin, but since these experiences generated by music are heavily associated with autobiographical memories therefore characterised by real loss as well, the putative hormonal response is unlikely to be converted into pleasure.

In a similar fashion, neural structures associated with different kinds of sadness experiences associated with music are bound to have different neural correlates. A clear demonstration of this was a study by Trost and her colleagues [69] who had participants listening to excerpts of classical music spanning nine target dimensions of emotions. The low arousal aesthetic emotions (transcendence, peacefulness, tenderness, and nostalgia) activated the right ventral striatum, hippocampus, and anterior cingulate and medial orbitofrontal cortex, whereas sadness itself displayed activations in right parahippocampal areas. Positive emotions have also been linked with social functions of music, and areas such as superficial amygdala associated with social signals [70]. An actual grief experience with acute psychological pain is known to activate rather a different set of brain areas, such as left cuneus and posterior cingulate cortex as well as posterior cingulate cortex, cerebellum, and parahippocampal gyrus [71]. An interesting follow-up to recent neuroimaging studies of music-induced sadness [12, 69] would be to differentiate the types of experiences based on mechanisms, reactions, and valenced descriptions of the experience and then dissociate the pertinent neural structures involved in these specific types of sadness experiences.

In sum, the discussion of all putative explanations of enjoyment of music-induced sadness has highlighted the need to differentiate the experiences associated with sadness: it is likely that different types of experiences are governed by different functions, mechanisms and explanations, as discussed above.

Finally, the current results detailing the emotions induced by music linked with sadness bear similarities to discoveries made in relation to the appeal of sadness in other forms of fiction [72, 73], and even with other negative emotions such as horror in films [74, 75] or disgust in visual art [76]. Overall, it seems that looking at underlying similarities between different types of fiction and focussing on a emotional reactions such as “being moved” [77] or “awe” or the experiences identified as *Sublime Sorrow* would be putting the notions of classical philosophy of aesthetics—as presented by Burke [78], Kant [79], Hume [80], and Schiller [81]—to much-awaited empirical scrutiny.

## Supporting Information

S1 Table Sample characteristics. (PDF)

S2 Table Proportion of participants selecting each reason for listening to sad music in each sample. (PDF)

S3 Table Proportions and rankings of the mechanisms relevant for memorable experience of sad music across the samples. (PDF)

## References

[1] Juslin PN, Liljeström S, Laukka P, Västfjäll D, Lundqvist LO. Emotional reactions to music in a nationally representative sample of Swedish adults Prevalence and causal influences. Musicae Scientiae. 2011;15(2):174–207. 10.1177/1029864911401169

[2] Bonnano GA, Goorin L, Coifman KC. Sadness and Grief In: Lewis MJ, Haviland-Jones M, Barrett LF, editors. Handbook of Emotions. 3rd ed. New York: The Guilford Press; 2008 p. 797–810.

[3] Eerola T, Vuoskoski JK. A comparison of the discrete and dimensional models of emotion in music. Psychology of Music. 2011;39(1):18–49. 10.1177/0305735610362821

[4] Garrido S, Schubert E. Moody melodies: Do they cheer us up? A study of the effect of sad music on mood. Psychology of Music. 2015;43(2):244–261. 10.1177/0305735613501938

[5] Kawakami A, Furukawa K, Katahira K, Okanoya K. Sad music induces pleasant emotion. Emotion Science. 2013;4:311. 10.3389/fpsyg.2013.00311 PMC3682130 23785342

[6] Taruffi L, Koelsch S. The paradox of music-evoked sadness: An online survey. PLoS ONE. 2014 10;9(10):e110490 10.1371/journal.pone.0110490 25330315 PMC4203803

[7] Barrett FS, Grimm KJ, Robins RW, Wildschut T, Sedikides C, Janata P. Music-evoked nostalgia: affect, memory, and personality. Emotion. 2010;10(3):390–403. 10.1037/a0019006 20515227

[8] Van den Tol AJM, Edwards J. Listening to sad music in adverse situations: Music selection strategies, self-regulatory goals, listening effect, and mood-enhancement. Psychology of Music. 2015;43(4):473–494. 10.1177/0305735613517410

[9] Vuoskoski JK, Thompson B, McIlwain D, Eerola T. Who enjoys listening to sad music and why? Music Perception. 2012;29(3):311–317.

[10] Vuoskoski JK, Eerola T. Can sad music really make you sad? Indirect measures of affective states induced by music and autobiographical memories. Psychology of Aesthetics, Creativity, and the Arts. 2012;6(3):204–213. 10.1037/a0026937

[11] Juslin PN, Harmat L, Eerola T. What makes music emotionally significant? Exploring the underlying mechanisms. Psychology of Music. 2013;42(4):599–623. 10.1177/0305735613484548

[12] Brattico E, Bogert B, Alluri V, Tervaniemi M, Eerola T, Jacobsen T. It’s sad but I like it: The neural dissociation between musical emotions and liking in experts and laypersons. Frontiers in Human Neuroscience. 2016;9(6). 10.3389/fnhum.2015.00676 26778996 PMC4701928

[13] Bodner E, Fradkin D. Tearful oriental songs and the role they play among adolescents. Psychology of Music. 2013;41(3):329–349. 10.1177/0305735611425905

[14] Agawu VK. Music in the funeral traditions of the Akpafu. Ethnomusicology. 1988;32(1):75–105. 10.2307/852226

[15] Mills S. Sounds to soothe the soul: Music and bereavement in a traditional South Korean death ritual. Mortality. 2012;17(2):145–157. 10.1080/13576275.2012.675231

[16] Briggs CL. Personal sentiments and polyphonic voices in Warao women’s ritual wailing: music and poetics in a critical and collective discourse. American Anthropologist. 1993;95(4):929–957. 10.1525/aa.1993.95.4.02a00080

[17] Meyer RK, Palmer C, Mazo M. Affective and coherence responses to Russian laments. Music Perception. 1998;16(1):135–150. 10.2307/40285782

[18] Peltola HR, Eerola T. Fifty Shades of Blue: Classification of music-evoked sadness. Musicae Scientiae. 2016;20(1):84–102. 10.1177/1029864915611206

[19] Van den Tol AJM, Edwards J. Exploring a rationale for choosing to listen to sad music when feeling sad. Psychology of Music. 2013;41(4):440–465. 10.1177/0305735611430433

[20] Robinson MD, Clore GL. Episodic and semantic knowledge in emotional self-report: Evidence for two judgment processes. Journal of Personality and Social Psychology. 2002;83:198–215. 10.1037/0022-3514.83.1.198 12088126

[21] Zentner M, Grandjean D, Scherer K. Emotions evoked by the sound of music: Characterization, classification, and measurement. Emotion. 2008;8(4):494–521. 10.1037/1528-3542.8.4.494 18729581

[22] Eerola T, Peltola HR, Vuoskoski JK. Attitudes towards sad music are related to both preferential and contextual strategies. Psychomusicology: Music, Mind, and Brain. 2015;25(2):116–123. 10.1037/pmu0000096

[23] Vingerhoets AJJM, Cornelius RR. Adult crying inventory In: Vingerhoets AJJM, Cornelius RR, editors. Adult crying. A biopsychosocial approach. New York: Taylor & Francis; 2001 p. 303–316.

[24] Juslin PN. From everyday emotions to aesthetic emotions: Toward a unified theory of musical emotions. Physics of Life Reviews. 2013;10(3):235–266. 10.1016/j.plrev.2013.05.008 23769678

[25] van der Eijk C, Rose J. Risky business: factor analysis of survey data—assessing the probability of incorrect dimensionalisation. PLoS ONE. 2015;10(3):e0118900 10.1371/journal.pone.0118900 25789992 PMC4366083

[26] Velicer WF. Determining the number of components from the matrix of partial correlations. Psychometrika. 1976;41(3):321–327. 10.1007/BF02293557

[27] McRae K, Ochsner KN, Mauss IB, Gabrieli JJD, Gross JJ. Gender differences in emotion regulation: An fMRI study of cognitive reappraisal. Group Processes & Intergroup Relations. 2008;11(2):143–162. 10.1177/1368430207088035 29743808 PMC5937254

[28] Zwick WR, Velicer WF. Comparison of five rules for determining the number of components to retain. Psychological Bulletin. 1986; (99):432–442. 10.1037/0033-2909.99.3.432

[29] Huron D. Why is sad music pleasurable? A possible role for prolactin. Musicae Scientiae. 2011;15(2):146–158. 10.1177/1029864911401171

[30] Juslin PN, Västfjäll D. Emotional responses to music: The need to consider underlying mechanisms. Behavioral and Brain Sciences. 2008;31(5):559–575. 10.1017/S0140525X08005293 18826699

[31] Braun V, Clarke V. Using thematic analysis in psychology. Qualitative Research in Psychology. 2006;3(2):77–101. 10.1191/1478088706qp063oa

[32] Guba EG, Lincoln YS. Competing paradigms in qualitative research In: Denzin NK, Lincoln YS, editors. Handbook of qualitative research. Thousand Oaks, CA: Sage; 1994 p. 105–117.

[33] Smith JM, Alloy LB. A roadmap to rumination: A review of the definition, assessment, and conceptualization of this multifaceted construct. Clinical Psychology Review. 2009;29(2):116–128. 10.1016/j.cpr.2008.10.003 19128864 PMC2832862

[34] Peter M, Vingerhoets AJJM, Van Heck GL. Personality, gender, and crying. European Journal of Personality. 2001;15(1):19–28. 10.1002/per.386

[35] Hemert DAv, Vijver FJRvd, Vingerhoets AJJM. Culture and Crying Prevalences and Gender Differences. Cross-Cultural Research. 2011;45(4):399–431. 10.1177/1069397111404519

[36] Vuoskoski JK, Eerola T. Measuring music-induced emotion: A comparison of emotion models, personality biases, and intensity of experiences. Musicae Scientiae. 2011;15(2):159–173. 10.1177/1029864911403367

[37] Warriner AB, Kuperman V, Brysbaert M. Norms of valence, arousal, and dominance for 13,915 English lemmas. Behavior Research Methods. 2013;45(4):1191–1207. 10.3758/s13428-012-0314-x 23404613

[38] Fan X, Sivo SA. Sensitivity of fit indices to model misspecification and model types. Multivariate Behavioral Research. 2007;42(3):509–529. 10.1080/00273170701382864

[39] Russell JA. A circumplex model of affect. Journal of Personality and Social Psychology. 1980;39(6):1161–1178. 10.1037/h0077714

[40] Rosseel Y. Lavaan: An R package for structural equation modeling. Journal of Statistical Software. 2012;48(2):1–36. 10.18637/jss.v048.i02

[41] R Core Team. R: A Language and Environment for Statistical Computing. Vienna, Austria; 2015 Available from: https://www.R-project.org/.

[42] Stevens JS, Hamann S. Sex differences in brain activation to emotional stimuli: a meta-analysis of neuroimaging studies. Neuropsychologia. 2012;50(7):1578–1593. 10.1016/j.neuropsychologia.2012.03.011 22450197

[43] Saarikallio SH. Music in mood regulation: Initial scale development. Musicae Scientiae. 2008;12(2):291–309. 10.1177/102986490801200206

[44] Baron-Cohen S, Wheelwright S, Hill J, Raste Y, Plumb I. The “Reading the Mind in the Eyes” test revised version: A study with normal adults, and adults with Asperger syndrome or high-functioning autism. Journal of Child Psychology and Psychiatry. 2001;42(2):241–251. 10.1111/1469-7610.00715 11280420

[45] Wager TD, Phan KL, Liberzon I, Taylor SF. Valence, gender, and lateralization of functional brain anatomy in emotion: a meta-analysis of findings from neuroimaging. Neuroimage. 2003;19(3):513–531. 10.1016/S1053-8119(03)00078-8 12880784

[46] Smith A. Cognitive empathy and emotional empathy in human behavior and evolution. The Psychological Record. 2006;56(1):3–21.

[47] Christov-Moore L, Simpson EA, Coudé G, Grigaityte K, Iacoboni M, Ferrari PF. Empathy: gender effects in brain and behavior. Neuroscience & Biobehavioral Reviews. 2014;46:604–627. 10.1016/j.neubiorev.2014.09.001 25236781 PMC5110041

[48] Thompson AE, Voyer D. Sex differences in the ability to recognise non-verbal displays of emotion: A meta-analysis. Cognition and Emotion. 2014;28(7):1164–1195. 10.1080/02699931.2013.875889 24400860

[49] Tibshirani R. Regression shrinkage and selection via the lasso. Journal of the Royal Statistical Society. 1996;58:267–288.

[50] Landgrebe TC, Duin RP. Approximating the multiclass ROC by pairwise analysis. Pattern recognition letters. 2007;28(13):1747–1758. 10.1016/j.patrec.2007.05.001

[51] Juslin PN, Liljeström S, Västfjäll D, Barradas G, Silva A. An experience sampling study of emotional reactions to music: Listener, music, and situation. Emotion. 2008;8(5):668–683. 10.1037/a0013505 18837617

[52] Randall WM, Rickard NS, Vella-Brodrick DA. Emotional outcomes of regulation strategies used during personal music listening: A mobile experience sampling study. Musicae Scientiae. 2014;18(3):275–291. 10.1177/1029864914536430

[53] Garrido S, Schubert E. Negative emotion in music: what is the attraction? A qualitative study. Empirical Musicology Review. 2011;6(4).

[54] Kotthoff H. Aesthetic dimensions of Georgian grief rituals: on the artful display of emotions in lamentation In: Knoblauch H, Kotthoff H, editors. Verbal art across cultures. The Aesthetics and Proto-Aesthetics of Communication. Tübingen: Narr; 2001 p. 167–194.

[55] Juslin PN. What does music express? Basic emotions and beyond. Emotion Science. 2013;4:596. 10.3389/fpsyg.2013.00596 PMC3764399 24046758

[56] Gabrielsson A. Strong experiences with music: Music is much more than just music. Oxford University Press; 2011.

[57] Gabrielsson A, Lindström E. The role of structure in the musical expression of emotions Handbook of music and emotion: Theory, research, applications 2010; p. 367–400.

[58] Zentner MR, Eerola T. Self-report measures and models In: Juslin PN, Sloboda JA, editors. Handbook of Music and Emotion. Boston, MA: Oxford University Press; 2010 p. 187–221.

[59] Eerola T, Vuoskoski JK, Kautiainen H. Being Moved by Unfamiliar Sad Music is Associated with High Empathy. Frontiers in Human Neuroscience. submitted;. 10.3389/fpsyg.2016.01176 PMC5025521 27695424

[60] Garrido S, Schubert E. Individual differences in the enjoyment of negative emotion in music: a literature review and experiment. Music Perception: An Interdisciplinary Journal. 2011;28(3):279–296. 10.1525/mp.2011.28.3.279

[61] Bigand E, Vieillard S, Madurell F, Marozeau J, Dacquet A. Multidimensional scaling of emotional responses to music: The effect of musical expertise and of the duration of the excerpts. Cognition & Emotion. 2005;19(8):1113–1139. 10.1080/02699930500204250

[62] Carstensen LL, Pasupathi M, Mayr U, Nesselroade JR. Emotional experience in everyday life across the adult life span. Journal of Personality and Social Psychology. 2000;79(4):644–655. 10.1037/0022-3514.79.4.644 11045744

[63] Sachs M, Damasio A, Habibi A. The Pleasures of Sad Music: A Systematic Review. Frontiers in Human Neuroscience. 2015;9(404). 10.3389/fnhum.2015.00404 26257625 PMC4513245

[64] Kivy P. Music alone: Philosophical reflections on the purely musical experience. Cornell University Press; 1990.

[65] Schönfelder S, Kanske P, Heissler J, Wessa M. Time course of emotion-related responding during distraction and reappraisal. Social Cognitive and Affective Neuroscience. 2014;9(9):1310–1319. 10.1093/scan/nst116 23988760 PMC4158366

[66] Drake JE, Winner E. How children use drawing to regulate their emotions. Cognition & Emotion. 2013;27(3):512–520. 10.1080/02699931.2012.720567 22963448

[67] Mar RA, Oatley K. The function of fiction is the abstraction and simulation of social experience. Perspectives on Psychological Science. 2008;3(3):173–192. 10.1111/j.1745-6924.2008.00073.x 26158934

[68] Turner RA, Altemus M, Yip DN, Kupferman E, Fletcher D, Bostrom A, et al Effects of emotion on oxytocin, prolactin, and ACTH in women. Stress: The International Journal on the Biology of Stress. 2002;5(4):269–276. 10.1080/1025389021000037586-1 12475731

[69] Trost W, Ethofer T, Zentner M, Vuilleumier P. Mapping aesthetic musical emotions in the brain. Cerebral Cortex. 2012;22(12):2769–2783. 10.1093/cercor/bhr353 22178712 PMC3491764

[70] Koelsch S, Skouras S, Fritz T, Herrera P, Bonhage C, Küssner MB, et al The roles of superficial amygdala and auditory cortex in music-evoked fear and joy. NeuroImage. 2013;81:49–60. 10.1016/j.neuroimage.2013.05.008 23684870

[71] Meerwijk EL, Ford JM, Weiss SJ. Brain regions associated with psychological pain: implications for a neural network and its relationship to physical pain. Brain Imaging and Behavior. 2013;7(1):1–14. 10.1007/s11682-012-9179-y 22660945

[72] Knobloch-Westerwick S, Gong Y, Hagner H, Kerbeykian L. Tragedy viewers count their blessings feeling low on fiction leads to feeling high on life. Communication Research. 2013;40(6):747–766. 10.1177/0093650212437758

[73] Goldstein TR. The pleasure of unadulterated sadness: Experiencing sorrow in fiction, nonfiction, and “in person”. Psychology of Aesthetics, Creativity, and the Arts. 2009;3(4):232–237. 10.1037/a0015343

[74] Goldstein J. The attractions of violent entertainment. Media Psychology. 1999;1:271–282. 10.1207/s1532785xmep0103_5

[75] Tamborini R, Stiff J. Predictors of horror film attendance and appeal an analysis of the audience for frightening films. Communication Research. 1987;14(4):415–436. 10.1177/009365087014004003

[76] Wagner V, Menninghaus W, Hanich J, Jacobsen T. Art schema effects on affective experience: The case of disgusting images. Psychology of Aesthetics, Creativity, and the Arts. 2014;8:120–129. 10.1037/a0036126

[77] Hanich J, Wagner V, Shah M, Jacobsen T, Menninghaus W. Why we like to watch sad films. The pleasure of being moved in aesthetic experiences. Psychology of Aesthetics, Creativity, and the Arts. 2014;8(2):130–143. 10.1037/a0035690

[78] Edmund B. A Philosophical Enquiry into the Origin of our Ideas of the Sublime and Beautiful. London, R. and J. Dodsley; 1757.

[79] Kant I. Critique of the Power of Judgment. Guyer P, editor. Cambridge: Cambridge University Press; 1790/2000.

[80] Hume D. Of Tragedy. A. Millar; 1757.

[81] Schiller F. Of the Cause of the Pleasure We Derive from Tragic Objects. Aesthetical and Philosophical Essays. 1792;2:86–99.

