# Supplementary material for: Memorable Experiences with Sad Music—Reasons, Reactions and Mechanisms of Three Types of Experiences
Source: PLoS One. 2016 Jun 14;11(6):e0157444. doi: 10.1371/journal.pone.0157444 (PMC4907454; doi:10.1371/journal.pone.0157444)
Supplement: S1 Table — (PDF) [file pone.0157444.s001.pdf]

PLOS

Eerola. T., & Peltola, H.-R. (2016). Memorable Experiences with Sad Music – Reasons, Reactions and Mechanisms of Three Types of Experiences. Plos One.

S1 Table

Sample characteristics.

	S1		S2		S3	
Total N	1577		445		414	
**Gender**	Women	Men	Women	Men	Women	Men
N	1171	406	237	208	209	205
%	74.3	25.7	53.3	46.7	50.5	49.5
**Age**						
18 to 24 (%)	23.0	21.4	11.4	6.7	25.4	13.2
25 to 34 (%)	30.8	33.7	20.7	13.5	25.8	33.2
35 to 44 (%)	19.7	24.9	23.6	21.2	21.1	22.4
45 to 54 (%)	13.5	13.5	22.8	28.8	15.3	16.1
55 to 64 (%)	10.1	4.7	19.8	26.0	9.6	10.2
65 to 74 (%)	2.9	1.7	1.7	2.9	2.9	4.9
**Listen to sad music**						
Frequently (%)	17.3	36.2	10.1	8.65	8.61	10.7
Often (%)	30.1	44.8	17.3	23.6	21.1	19.5
Sometimes (%)	45.8	18.2	46.4	43.3	46.9	46.8
Rarely (%)	5.6	0.7	17.7	13.0	18.2	18.0
Never (%)	1.2	0.0	8.4	11.5	5.3	4.9
