# Supplementary material for: Memorable Experiences with Sad Music—Reasons, Reactions and Mechanisms of Three Types of Experiences
Source: PLoS One. 2016 Jun 14;11(6):e0157444. doi: 10.1371/journal.pone.0157444 (PMC4907454; doi:10.1371/journal.pone.0157444)
Supplement: S2 Table — (PDF) [file pone.0157444.s002.pdf]

Eerola. T., & Peltola, H.-R. (2016). Memorable Experiences with Sad Music – Reasons, Reactions and Mechanisms of Three Types of Experiences. Plos One.

## S2 Table

**Proportion of participants selecting each reason for listening to sad music in each sample.**

| **Reason (I listen to sad music ...)** | **S1** | **S2** | **S3** |
|---|---|---|---|
| ... because I find it beautiful | 86.6 | 38.4 | 72.5 |
| ... when I am able to listen to it privately | 78.6 | 45.6 | 75.1 |
| ... to get comfort (music as a supportive friend) | 68.4 | 28.3 | 57.7 |
| ... to empathize with the expressive qual. of music | 65.9 | 14.6 | 50.2 |
| ... to reminisce about past events, places or people | 65.9 | 40.7 | 63.8 |
| ... to sort my thoughts | 62.1 | 24.3 | 52.7 |
| ... to channel my emotions | 62.0 | 24.3 | 50.5 |
| ... to calm down and relax | 61.9 | 32.4 | 55.6 |
| ... to match my emotional state with the music | 58.9 | 14.4 | 47.8 |
| ... to empathize with the story conveyed by music | 54.0 | 22.9 | 42.8 |
| ... to get in touch with my emotions | 53.6 | 23.8 | 43.2 |
| ... to re-experience feelings I have experienced before | 50.6 | 18.2 | 44.7 |
| ... to enhance my mood | 37.1 | 11.7 | 30.9 |
| ... to share my feelings induced by music with others | 35.4 | 15.3 | 23.2 |
| ... to experience new feelings | 24.5 | 7.6 | 17.9 |
| ... to feel closer to my loved ones | 23.1 | 13.9 | 21.5 |
| ... to reveal my musical preferences to others | 22.7 | 9.9 | 17.1 |
| ... to feel connected with the others | 20.7 | 16.4 | 15.0 |
| ... to get more realistic perspective on life | 18.5 | 12.6 | 14.7 |
| ... to let the music express thoughts to others | 18.3 | 12.8 | 12.8 |
| ... to experience sense of belonging to a community | 10.0 | 5.6 | 7.5 |
| ... because of other reasons | 7.9 | 11.2 | 8.5 |
| ... to distract myself of feeling unwanted emotions | 6.3 | 8.8 | 5.1 |
| ... when I have company while listening to sad music | 1.6 | 7.9 | 2.2 |
