# Supplementary material for: Memorable Experiences with Sad Music—Reasons, Reactions and Mechanisms of Three Types of Experiences
Source: PLoS One. 2016 Jun 14;11(6):e0157444. doi: 10.1371/journal.pone.0157444 (PMC4907454; doi:10.1371/journal.pone.0157444)
Supplement: S3 Table — (PDF) [file pone.0157444.s003.pdf]

Eerola. T., & Peltola, H.-R. (2016). Memorable Experiences with Sad Music – Reasons, Reactions and Mechanisms of Three Types of Experiences. Plos One.

S3 Table

Proportions and rankings of the mechanisms relevant for memorable experience of sad music across the samples.

S1	S2	S3	Mechanism	Component
79.5	28.5	71.5	Emotion expressed	Aesthetic
67.3	26.3	58.5	Beauty of music	Aesthetic
46.6	26.1	43.5	Associations and meanings	Aesthetic
45.0	39.6	45.2	Memories	Memory
41.2	12.6	34.5	Visual imagery	-
17.2	6.5	15.5	Re-experiencing the emotion	Memory
16.6	13.0	15.5	Strong, captivating rhythm	Music
14.0	11.2	13.3	Sharing emotions	Aesthetic
12.2	8.3	13.5	Expectations about music	Memory
7.2	6.1	7.0	Surprising event	Music
